# Bramwell-Hill modeling for local aortic pulse wave velocity estimation: a validation study with velocity-encoded cardiovascular magnetic resonance and invasive pressure assessment

**DOI:** 10.1186/1532-429X-14-2

**Published:** 2012-01-09

**Authors:** Jos JM Westenberg, Eveline P van Poelgeest, Paul Steendijk, Heynric B Grotenhuis, JW Jukema, Albert de Roos

**Affiliations:** 1 Department of Radiology, Leiden University Medical Center, Leiden, The Netherlands; 2 Department of Internal Medicine, Leiden University Medical Center, Leiden, The Netherlands; 3 Department of Cardiology, Leiden University Medical Center, Leiden, The Netherlands

## Abstract

**Background:**

The Bramwell-Hill model describes the relation between vascular wall stiffness expressed in aortic distensibility and the pulse wave velocity (PWV), which is the propagation speed of the systolic pressure wave through the aorta. The main objective of this study was to test the validity of this model locally in the aorta by using PWV-assessments based on in-plane velocity-encoded cardiovascular magnetic resonance (CMR), with invasive pressure measurements serving as the gold standard.

**Methods:**

Seventeen patients (14 male, 3 female, mean age ± standard deviation = 57 ± 9 years) awaiting cardiac catheterization were prospectively included. During catheterization, intra-arterial pressure measurements were obtained in the aorta at multiple locations 5.8 cm apart. PWV was determined regionally over the aortic arch and locally in the proximal descending aorta. Subsequently, patients underwent a CMR examination to measure aortic PWV and aortic distention. Distensibility was determined locally from the aortic distension at the proximal descending aorta and the pulse pressure measured invasively during catheterization and non-invasively from brachial cuff-assessment. PWV was determined regionally in the aortic arch using through-plane and in-plane velocity-encoded CMR, and locally at the proximal descending aorta using in-plane velocity-encoded CMR. Validity of the Bramwell-Hill model was tested by evaluating associations between distensibility and PWV. Also, theoretical PWV was calculated from distensibility measurements and compared with pressure-assessed PWV.

**Results:**

In-plane velocity-encoded CMR provides stronger correlation (p = 0.02) between CMR and pressure-assessed PWV than through-plane velocity-encoded CMR (r = 0.69 versus r = 0.26), with a non-significant mean error of 0.2 ± 1.6 m/s for in-plane versus a significant (p = 0.006) error of 1.3 ± 1.7 m/s for through-plane velocity-encoded CMR. The Bramwell-Hill model shows a significantly (p = 0.01) stronger association between distensibility and PWV for local assessment (r = 0.8) than for regional assessment (r = 0.7), both for CMR and for pressure-assessed PWV. Theoretical PWV is strongly correlated (r = 0.8) with pressure-assessed PWV, with a statistically significant (p = 0.04) mean underestimation of 0.6 ± 1.1 m/s. This theoretical PWV-estimation is more accurate when invasively-assessed pulse pressure is used instead of brachial cuff-assessment (p = 0.03).

**Conclusions:**

CMR with in-plane velocity-encoding is the optimal approach for studying Bramwell-Hill associations between local PWV and aortic distensibility. This approach enables non-invasive estimation of local pulse pressure and distensibility.

## Background

The pathophysiological processes of cardiovascular disease involve stiffening of the arterial vessel wall. Increased aortic wall stiffness results in an increased aortic pulse pressure and left ventricular afterload, restricting left ventricular filling during diastole, which eventually may lead to heart failure [1,2]. Additionally, aortic stiffening is an important risk factor for end organ damage with coronary, renal or cerebral expression as the hemodynamic load on the end organs is increased with impaired damping of the systolic wave [3-10]. The growing awareness of the prognostic value of aortic stiffness for the prediction of cardiovascular morbidity and mortality, as highlighted in a meta-analysis published by Vlachopoulos et al. [11], increases the recognition of stiffness-assessment as a surrogate end point for cardiovascular disease in clinical research [12].

Assessment of regional PWV is of high interest in cardiovascular research as the independent prognostic value of regional PWV-assessment for outcome prediction in various patient populations has been recognized [2-4,7-10]. Since the majority of the reservoir capacity of the arterial system resides in the proximal part of the aorta, stiffness assessment in this region will provide essential information on the aortic condition and function. Moreover, as the aorta changes in structure over its length considerably with age, regional identification of increased wall stiffening may provide valuable insight into the underlying pathology.

Several estimators - such as Young's modulus, distensibility or stiffness index - are currently in use to express aortic stiffness, all relating local blood pressure with the distention of the aorta (either by diameter or luminal area). Aortic distensibility is defined as the relative change in vessel diameter over local pulse pressure [13,14]. A useful surrogate marker of aortic stiffness is the pulse wave velocity (PWV), which is defined as the velocity of the systolic pulse wave front propagating through the aorta. The PWV is increased when atherosclerotic wall degeneration and concomitant reduction of elastic recoil are present, and PWV has proven to be an independent and strong predictor of cardiovascular morbidity and mortality [15-19].

The gold standard for PWV-assessment is defined from invasive pressure measurements at consecutive locations in the aorta, from which the propagation speed of the systolic pressure wave front can be accurately determined. Cardiovascular Magnetic Resonance (CMR) with velocity-encoding (VE) is a validated alternative for measuring the PWV [20], globally for the whole aorta, regionally in specific aortic segments as well as locally at a specific position in the aorta [21].

The Bramwell-Hill model [22] theoretically links PWV, aortic distensibility and pulse pressure together. This model is derived from the Moens-Korteweg equation which, under modeling assumptions (i.e. vessel wall thickness is small compared to the diameter and the circulating fluid within the vessel is incompressible and nonviscous), relates arterial stiffness and PWV. Recently, Dogui et al. [23] tested the validity of this model and the associations between pulse pressure, PWV and aortic distensibility using VE CMR regionally in the aortic arch. However, in their experiments, no PWV or pulse pressure information was available locally in the aorta, whereas distensibility is an intrinsic local measure. In our present study, we aim to overcome the shortcomings of the study of Dogui et al. by using local PWV-assessment from in-plane VE CMR. Furthermore, invasive pressure measurements are obtained during catheterization to determine the local pulse pressure for accurate distensibility assessments, and to determine the gold standard for PWV-assessment. Therefore, the main objective of this study was to test the validity of the Bramwell-Hill model locally in the aorta by using PWV-assessments based on in-plane VE CMR-acquisitions, with invasive pressure measurements as the gold standard.

## Methods

### Subjects

Data from the subjects reported in this study have been described earlier in studies validating PWV-assessment with VE-CMR [20,21]. Research was carried out in compliance with the Helsinki Declaration. The study was approved by the local Medical Ethical Committee. A total of 17 patients (14 male and 3 female, mean age ± standard deviation (SD) = 57 ± 9 years) with suspected coronary artery disease awaiting elective cardiac catheterization were prospectively enrolled in the study after giving informed consent.

Patients underwent CMR examination to measure aortic PWV and aortic distention. The mean interval between catheterization and CMR was 17 ± 13 days. Exclusion criteria were general contraindications to CMR, evidence of aortic valve stenosis on ultrasonography, coarctation of the aorta, or other congenital heart disease, or a family history of Marfan syndrome.

### Theoretical modeling

PWV is defined as the distance traveled (Δ*x*) by a wave (pressure or blood flow) divided by the transit-time (Δ*t*) for the wave to travel that distance: PWV = Δx/Δt. This definition holds true under the assumption that no wave reflections occur, as the transmission of the pressure pulse as a sum of incident and reflected waves does not represent the true PWV [24]. Alternatively, PWV may be assessed using the foot-to-foot method on pressure or flow wave front propagation to calculate the transit-time, with only minimal interference of wave reflections [12]. The Bramwell-Hill model is derived from the Moens-Korteweg equation, linking PWV, vessel strain, pulse pressure and blood density [22] as follows:

(1) $$\text{PWV = (}\rho \times {\text{Distensibility)}}^{\text{ - 1/2}}$$

with ρ being the blood density (1059 kg∙m^-3^) and Distensibility defined by dV/(V∙dP), with V the aortic volume and P the blood pressure. Distensibility can be estimated by the relative luminal area change over the local pulse pressure:

(2) $$\text{Distensibility = }∆\text{A / (}{\text{A}}_{\text{minimal}}\;\times \;∆\text{P)}$$

with A being the luminal area and ΔA and ΔP the difference between maximal and minimal luminal area and blood pressure, respectively, during the cardiac cycle.

The relation between PWV, pulse pressure and distensibility will be tested by PWV-assessment from both invasive pressure measurements (gold standard) and VE CMR, with blood pressure assessment both invasively and from brachial cuff measurement, and assessment of aortic luminal area distention with CMR.

### Invasive pressure measurements for PWV_pressure_

Invasive pressure-time curves and simultaneous ECG recordings were obtained immediately after vascular access, to avoid any interference by medication or performed procedures. A 6F JR4 pressure tip catheter (Cordis, Miami Lakes, FL) was introduced through a 6F sheet (Cordis) into either one of the femoral arteries and advanced through the aorta until just distal to the aortic valve. During stepwise pullback, pressure waves were recorded at consecutive positions spaced 5.8 cm apart. Pressure-time curves and ECGs were recorded with a sampling resolution of 2 kHz during at least 10 cardiac cycles at each position to take into account variations induced by respiration. PWV_pressure _is calculated as Δx/Δt (expressed in m/s), where Δx is the aortic path length between measurement sites, determined from catheter pullback, and Δt is the transit-time for the systolic pressure wave front to propagate between these sites. The onset of the systolic pressure wave front was automatically determined from the time point (relative to the R-wave) of minimal pressure prior to the upslope of the systolic pressure wave. Local pulse pressure was determined from the difference between maximal and minimal pressure. Offline analysis of the pressure-time curves was performed using custom-made software.

### VE CMR for PWV_t.p. _and PWV_i.p._

**Figure 1:**
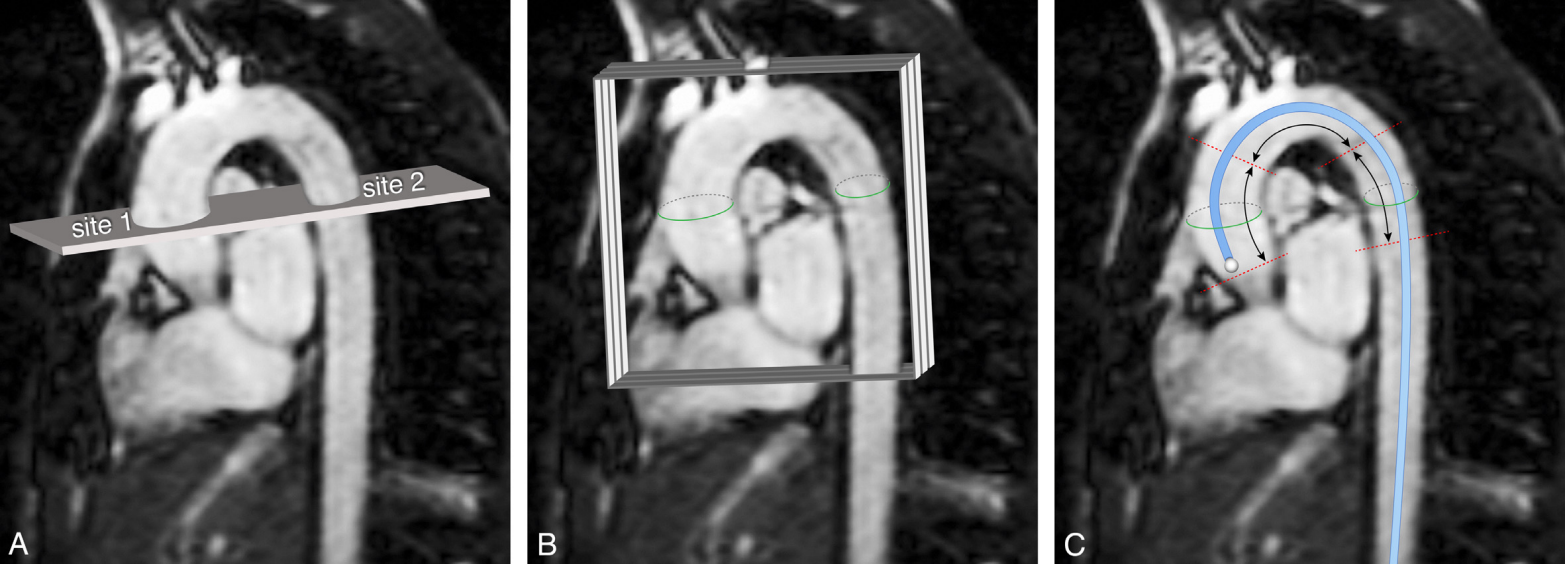
**Three methods for Pulse Wave Velocity-assessment**. A: PWV_t.p._: a CMR acquisition plane is positioned perpendicular to the ascending aorta, transecting both the ascending (site 1) and descending (site 2) aorta. Velocity is encoded perpendicular to the acquisition plane. Transit-time is determined for the systolic velocity wave front to propagate from site 1 to site 2. PWV_t.p. _is determined from the ratio of the distance between site 1 and 2 and this transit-time. B: PWV_i.p._: three consecutive CMR acquisition planes capture the aortic arch in double-oblique sagittal orientation. Velocity is encoded in-plane in two directions. The velocity propagation along the centerline of the aorta determines PWV_i.p._. C: PWV_pressure_: a pressure tip catheter is inserted in the aorta and positioned at the aortic valve. During pullback, invasive pressure is determined at positions 5.8 cm apart. The propagation of the pressure wave determines PWV_pressure_.

VE CMR was performed on a 1.5 Tesla CMR scanner with a typical total acquisition time of 25 minutes (ACS-NT15 Intera, Philips Medical Systems, Best, The Netherlands; software release 11, Pulsar gradient system with amplitude 33 mT/m and 100 mT/m/ms slew rate, 0.33 ms rise time). PWV was assessed regionally for the aortic arch using a single one-directional through-plane VE acquisition (i.e. PWV_t.p._), planned perpendicular to the aorta and transecting both the ascending aorta (site 1 in Figure 1) and proximal descending aorta (site 2). Furthermore, PWV was determined from 2-directional in-plane VE CMR (PWV_i.p._), both regionally for the same aortic arch trajectory as well as locally in the aorta at the imaging location of site 2.

Imaging sequences were previously described [20,21]. In short, first a stack of three consecutive double-oblique-sagittal slices was obtained capturing the full aorta from aortic root, arch to abdominal aorta, using cine-gradient-echo with retrospective gating and steady-state free-precession. This stack of slices covering the aorta was used for planning VE CMR-acquisitions and to measure the aortic path length of the aortic arch trajectory. Next, a single-slice one-directional through-plane VE CMR-acquisition was performed perpendicular to the aorta at the level of the pulmonary trunk, transecting both the ascending and proximal descending aorta distal to the aortic arch. A five-element cardiac surface coil was used for signal reception. Scan parameters were: 90% rectangular FOV 300 × 270 mm^2^, 8 mm slice thickness, TE 2.9 msec, TR 4.9 msec, flip angle α 20°, acquisition voxel size 2.3 × 2.1 × 8.0 mm^3^, sampling bandwidth 449 Hz, number of signal averages 2, retrospective gating with maximal number of phases reconstructed into one average cardiac cycle with arrhythmia rejection window set to acceptance threshold of 15%-variation. The velocity sensitivity was set to 150 cm/s. Acquisition was performed with free breathing without respiratory compensation and scan time was almost 4 minutes at a heart rate of 60 bpm.

Finally, PWV_i.p. _was assessed by means of two consecutive multi-slice two-directional in-plane VE CMR-acquisitions of the three-slice double-oblique-sagittal stack of the aorta. VE was performed in phase-encoding (i.e., anterior-posterior AP) direction and in frequency-encoding (i.e., feet-head FH) direction respectively with velocity sensitivity set to 150 cm/s in either direction. The body coil was used for signal reception. Scan parameters were: 60%-rectangular FOV 450 × 270 mm^2^, 10 mm slice thickness, TE 2.4 ms, TR 4.3 ms, α 10°, acquisition voxel size 3.5 × 2.1 × 10.0 mm^3^, sampling bandwidth 495 Hz, NSA 2, retrospective-gating with maximal number of phases reconstructed into one average cardiac cycle with arrhythmia rejection window set to acceptance of 15%-variation. Acquisition was performed with free breathing without respiratory compensation and scan time of a single acquisition amounted to 7 minutes 42 seconds at a heart rate of 60 bpm.

For CMR-assessed PWV, Δx/Δt was calculated with Δx the aortic path length between measurement sites and Δt the transit-time for the systolic velocity wave front to propagate between these sites. The aortic path length between the subsequent measurement sites in ascending and proximal descending aorta was manually assessed by drawing a poly-line along the centerline of the aortic arch within the scout images, using the in-house developed software package MASS (Leiden University Medical Center, Leiden, The Netherlands) [25]. At each measurement site, maximal velocity-time curves were determined and used to evaluate the systolic wave propagation. These velocity-time curves were determined from aortic velocity maps which were analyzed with the in-house developed analytic software package FLOW (Leiden University Medical Center, Leiden, The Netherlands) [26], using automated contour detection. The onset of the systolic wave front, required for transit-time calculation, was automatically determined from the intersection point of a horizontal line modeling the constant horizontal diastolic flow and a line modeling the systolic upslope in the velocity-time curve. This line was modeled by linear regression of the flow velocity values within 20% and 80% of the total range of values along the systolic upslope. This algorithm was also used for calculating the transit-time for the propagation of the maximal velocity wave front, obtained with in-plane VE CMR. PWV_i.p. _was obtained as described before [21], from the two three-slice acquisitions with two-directional in-plane VE CMR. The full aorta was manually segmented with one set of contours that was subsequently copied to all slices, all phases and to both AP and FH encoding series. The aortic centerline was automatically determined from the contour set and 200 equidistantly-spaced sampling chords perpendicular to the centerline were defined. For each pixel within the aorta, the velocity in the direction parallel along the centerline was constructed from the velocity components in AP and FH direction. The aortic flow velocity was sampled along each chord and the maximal velocity per chord was determined. For each slice, this resulted in 200 velocity-time curves which were used to determine the transit-time as described above.

PWV_t.p. _and PWV_i.p. _were determined regionally in the aortic arch between measurement sites 1 and 2 and PWV_i.p. _was determined also locally around site 2, for an 11.6 cm trajectory starting 5.8 cm above site 2 to 5.8 cm below site 2. The aortic luminal distension was determined at site 2 from the maximal and minimal cross-sectional lumen area measured as described above with the automated contour detection on the velocity-insensitive magnitude gradient-echo images. Immediately after VE CMR-examination, brachial cuff blood pressure was obtained using a semi-automated sphygmomanometer (Dinamap, Critikon, Tampa, FL, USA), with the subject remaining in supine position on the CMR-table. Regional PWV_pressure _was determined over the aortic arch, from pressure measurements between sample points closest to measurement sites 1 and 2. Furthermore, local PWV_pressure _was determined for an 11.6 cm trajectory around site 2.

### Statistical Analysis

Statistical analysis was performed using SPSS for Windows (v. 12.0.1; Chicago, IL). All data are presented as mean values ± one SD, unless stated otherwise.

Associations of PWV_t.p. _and PWV_i.p. _with PWV_pressure _were evaluated using the Pearson's correlation coefficient (r), while variation with PWV_pressure _was studied with coefficients of variation (COV; defined as the standard deviation of the differences between the two series of measurements divided by the mean of both measurements). Also mean unsigned error (with PWV_pressure _as reference standard) and 95%-confidence intervals (95%-CI) were calculated. Statistical significance of differences between correlation coefficients was tested by stepwise linear regression analysis with the gold standard and the interaction between the gold standard and the tested methods as predictors. The approach described by Bland and Altman [27] was followed to study systematic trends in differences. Validity of the Bramwell-Hill model was tested by evaluating the association between regional and local PWV with local distensibility, using the pulse pressure measured both with brachial cuff-assessment at CMR examination (i.e. PP_cuff_) and intra-arterially during catheterization (i.e. PP_cath_). Furthermore, the estimated theoretical PWV from distensibility assessment was compared to the gold standard PWV_pressure_. Statistical significance on all tests was indicated by p < 0.05.

## Results

Patient characteristics are presented in Table 1. Mean time span between catheterization and CMR examination was 17 ± 13 days. At catheterization, 6 patients underwent coronary intervention. In two patients medication with beta blocking agent was initiated after catheterization. For these patients, CMR was performed 2 and 19 days later. In three patients, medication with an angiotensin converting enzyme inhibitor was started after catheterization and in one subject, dosage of the angiotensin converting enzyme inhibitor was increased.

**Table 1:** Patient characteristics

	n	Mean ± SD	Range
**Male**	14		

**Female**	3		

**Age (years)**		57 ± 9	34 - 75

**Length (cm)**		173 ± 12	157 - 185

**Weight (kg)**		82 ± 11	62 - 100

**Body mass index (kg.m^-2^)**		27 ± 4	22 - 35

**Body surface area (m^2^)**		2.0 ± 0.2	1.7 - 2.2

**Systolic blood pressure (mmHg)**		129 ± 21	97 - 166

**Diastolic blood pressure (mmHg)**		76 ± 13	53 - 100

**NYHA class**		2.2 ± 1.0	

**class I**	5		
**II**	6		
**III**	4		
**IV**	2		

NYHA: New York Heart Association; n: number; SD: standard deviation

Regionally in the aortic arch, mean PWV from in-plane VE CMR was 6.7 ± 2.3 m/s, mean PWV from through-plane was 5.6 ± 1.2 m/s and mean PWV from invasive pressure measurements was 6.9 ± 1.5 m/s. Locally at site 2, mean PWV from in-plane VE CMR was 7.1 ± 2.6 m/s and from invasive pressure measurement 7.0 ± 1.6 m/s.

**Figure 2:**
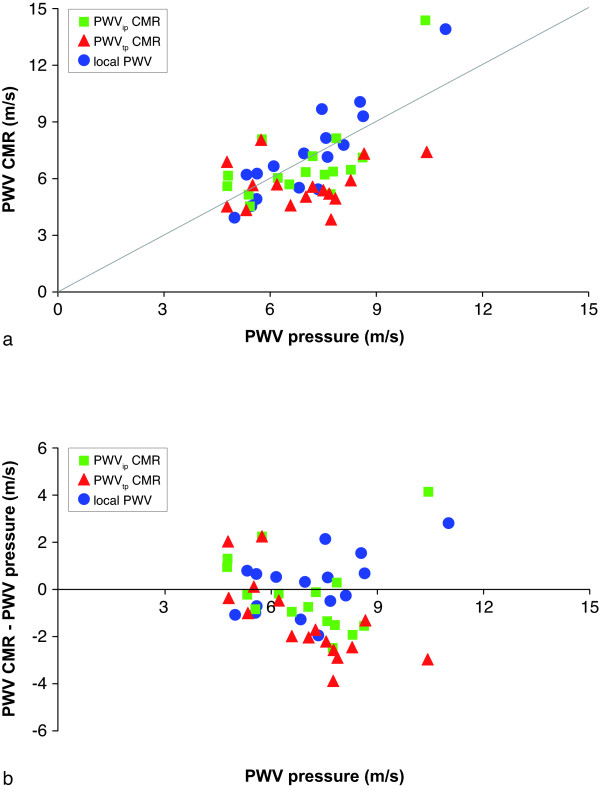
**Correlation between invasively-assessed PWV_pressure _and CMR-assessed PWV**. A: correlation between PWV_pressure _and CMR-assessed PWV regionally in the aortic arch from through-plane velocity-encoded CMR and in-plane VE CMR, and locally at the proximal descending aorta with in-plane VE CMR. B: Bland-Altman plot of the differences.

First, PWV_t.p. _and PWV_i.p. _regionally for the aortic arch and PWV_i.p. _locally at the proximal descending aorta were compared against PWV_pressure_. In Figure 2A, the correlation between CMR-assessed and pressure-assessed PWV are presented. In Figure 2B, the differences with the gold standard are presented in a Bland-Altman plot. Statistical results are summarized in Table 2. Correlation for regional PWV from through-plane VE CMR was low and not statistically significant (r = 0.26, p = 0.31), but improved (p = 0.02) for regional PWV-assessment from in-plane VE CMR (r = 0.69, p = 0.002). Variation with PWV_pressure _was high (27% and 24%, respectively). Mean unsigned error for PWV_t.p. _was 26% and for PWV_i.p. _18%. For local PWV-assessment, correlation was strong (r = 0.91, p < 0.001) and variation amounted to 19%. The mean unsigned error was 15%. Only regional PWV_t.p. _was significantly different from PWV_pressure _(mean underestimation of 1.3 ± 1.7 m/s, p = 0.006). From the Bland-Altman plot, it is clear that this underestimation increases with increasing PWV_pressure_.

**Table 2:** Associations between CMR- and pressure-assessed PWV.

	regional			local
	**PWV_t.p. _vs. PWV_pressure_**	***p-value comparison↔***	**PWV_i.p. _vs. PWV_pressure_**	**PWV_i.p. _vs. PWV_pressure_**

**Pearson r**	0.26 (p = 0.31)	0.02	0.69 (p = 0.002)	0.91 (p < 0.001)

**COV**	27%		24%	19%

**Mean unsigned error**	26% ± 13%		18% ± 12%	15% ± 8%

**Mean difference (m/s)**	-1.3 ± 1.7		-0.2 ± 1.6	0.2 ± 1.3

**p-value t-test**	0.006		0.61	0.56

**95%-CI (m/s)**	-2.1 - 0.5		-1.0 - 0.6	-0.4 - 0.8

PWV_t.p._: Pulse wave velocity from through-plane velocity-encoded CMR; PWV_i.p._: PWV from in-plane velocity-encoded CMR; PWV_pressure_: PWV from invasive pressure measurements; COV: coefficient of variation; 95%-CI: 95%-confidence interval.

Next, the validity of the Bramwell-Hill model was tested. Associations between all PWV-assessments (i.e. PWV_t.p._, PWV_i.p. _and PWV_pressure _regionally in the aortic arch and PWV_i.p. _and PWV_pressure _locally at the proximal descending aorta) with (local distensibility)^-1/2 ^(calculated with PP_cuff _and PP_cath_) are presented in Table 3. Local PWV-assessment showed stronger (all p ≤ 0.01) associations (r = 0.72, p = 0.001 for PWV_i.p. _with distensibility from PP_cuff _and r = 0.83, p < 0.001 for PWV_pressure _with distensibility from PP_cath_) with (distensibility)^-1/2 ^than regional PWV-assessment (PWV_i.p._: r = 0.63, p = 0.007 for PP_cuff _and r = 0.62, p = 0.007 for PP_cath_; PWV_t.p._: r = 0.40, p = 0.11 for PP_cuff _and r = 0.34, p = 0.18 for PP_cath_; PWV_pressure_: r = 0.45, p = 0.07 for PP_cuff _and r = 0.57, p = 0.02 for PP_cath_). Furthermore, PWV_pressure _showed strong correlation (r = 0.83, p < 0.001 for PP_cuff _and r = 0.80, p < 0.001 for PP_cath_) which was comparable (p ≥ 0.24) with CMR-assessed PWV (r = 0.72, p = 0.001 for PP_cuff _and r = 0.74, p = 0.001 for PP_cath_). None of the associations were significantly different when using PP-values from brachial cuff assessment or intra-arterially during catheterization.

**Table 3:** Association between PWV and (distensibility)^-1/2 ^according to Bramwell-Hill model.

	Distensibility with PP_cuff_	*p-value comparison* *↔*	Distensibility with PP_cath_
**regional PWV_t.p. _**	r = 0.40, p = 0.11	0.85	r = 0.34, p = 0.18

**regional PWV_i.p._**	r = 0.63, p = 0.007	0.49	r = 0.62, p = 0.007

***p-value comparison ↨***	0.01		0.01

**local PWV_i.p._**	r = 0.72, p = 0.001	0.33	r = 0.74, p = 0.001

***p-value comparison ↨***	0.30		0.24

**local PWV_pressure_**	r = 0.83, p < 0.001	0.27	r = 0.80, p < 0.001

***p-value comparison ↨***	< 0.001		< 0.001

**regional PWV_pressure_**	r = 0.45, p = 0.07	0.36	r = 0.57, p = 0.02

PWV_t.p._: Pulse wave velocity from through-plane velocity-encoded CMR; PWV_i.p._: PWV from in-plane velocity-encoded CMR; PWV_pressure_: PWV from invasive pressure measurements; PP_cuff_: pulse pressure from brachial cuff measurement; PP_cath_: pulse pressure from invasive pressure measurement during catheterization.

**Figure 3:**
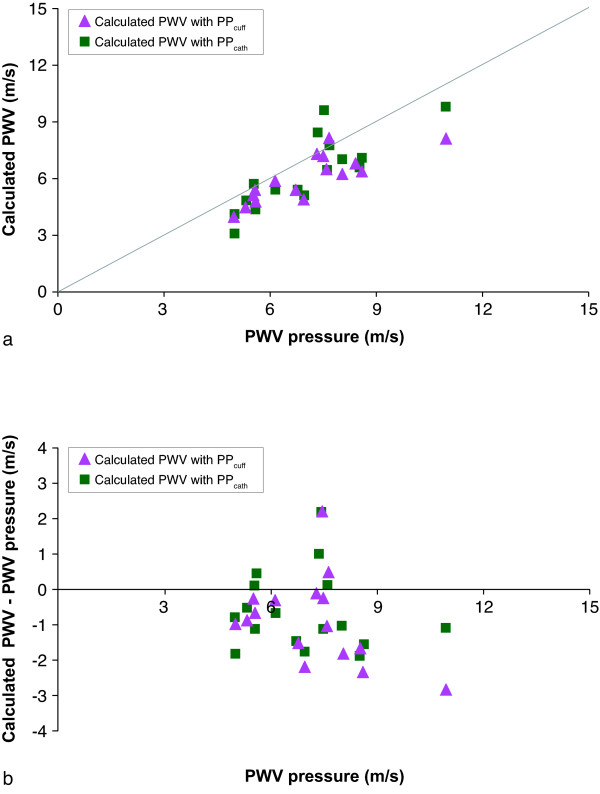
**Correlation between local PWV_pressure _and PWV calculated from aortic distensibility measurements**. A: correlation between local PWV from invasive pressure measurements (gold standard) and calculated PWV from aortic distensibility measurements with pulse pressure from brachial cuff and invasive pressure measurements during catheterization. B: Bland-Altman plot of the differences.

Finally, modeled local PWV-values were calculated from the measured local distensibility at the proximal descending aorta (with both PP_cuff _and PP_cath_). Correlations between these modeled local PWV-values and the gold standard (local PWV_pressure_) are presented in Figure 3A. Differences are presented in a Bland-Altman plot (Figure 3B). Statistical results are shown in Table 4. Correlation between modeled PWV-values and PWV_pressure _was strong (r = 0.82, p < 0.001 for PP_cuff _and r = 0.80, p < 0.001 for PP_cath_, respectively) and comparable (p = 0.27) for pressure measurements with brachial cuff and during catheterization, with COV of 14% for PP_cuff _and 17% for PP_cath _and mean unsigned errors of 16% for both PP_cuff _and PP_cath_. The Bland-Altman plot also shows a significant higher (p = 0.03) underestimation of the modeled PWV-values for PP_cuff _(mean underestimation 1.1 ± 0.9 m/s (p < 0.001)) than for PP_cath _(mean underestimation 0.6 ± 1.1 m/s (p = 0.04)).

**Table 4:** Association between PWV from Bramwell-Hill model (with brachial cuff and intra-arterial pressure) and pressure-assessed PWV.

	PP_cuff_	*p-value comparison* *↔*	PP_cath_
**Pearson r**	0.82 (p < 0.001)	0.27	0.80 (p < 0.001)

**COV**	14%		17%

**Mean unsigned error**	16% ± 9%		16% ± 9%

**Mean difference (m/s)**	-1.1 ± 0.9	0.03	-0.6 ± 1.1

**p-value t-test**	< 0.001		0.04

**95%-CI (m/s)**	-1.5 - -0.7		-1.1 - -0.1

PP_cuff_: pulse pressure from brachial cuff measurement; PP_cath_: pulse pressure from invasive pressure measurement during catheterization; COV: coefficient of variation; 95%-CI: 95%-confidence interval.

## Discussion

In this study, the validity of the Bramwell-Hill model - which describes the relation between pulse pressure, pulse wave velocity and distensibility locally in the aorta - was tested on velocity-encoded CMR-acquisitions with invasive pressure measurements serving as the gold standard. PWV was assessed with VE CMR as well as with invasive pressure measurements (i.e. the gold standard), both locally at the proximal descending aorta and regionally in the aortic arch. The main findings of our study are: 1) correlation between the gold standard and regional PWV-assessment in the aortic arch with in-plane VE CMR is significantly stronger than with through-plane VE CMR; 2) local PWV-assessment in the proximal descending aorta with in-plane VE CMR is stronger correlated with the gold standard than regional PWV-assessment in the aortic arch; 3) the Bramwell-Hill model shows stronger association between PWV, distensibility and pulse pressure when applied to local PWV-assessment as compared to regional assessment; 4) local PWV modeled from local distensibility is significantly correlated with the gold standard, but shows a significant underestimation, with a higher mean error for pulse pressure measurement from non-invasive brachial cuff than from invasive pressure assessment.

Many pathophysiological processes of cardiovascular diseases involve increased stiffening of the arterial vessel wall [1,2], which leads to abnormal wave propagation. Central aortic wall stiffening may be the starting point of a negative cascade of end organ damage and has been reported as an important risk factor for various expressions of end organ damage and dysfunction [3-10]. The Bramwell-Hill model describes the relation between PWV, distensibility and pulse pressure. Modeling assumptions on arterial vessel wall and flowing blood properties need to be taken into account, which implies that this model is not generally valid for all individuals. Furthermore, whereas distensibility and pulse pressure are assessed locally in the aorta, local PWV can only be estimated since it is determined from the wave propagation over a particular aortic trajectory, with the optimal estimation from in-plane VE CMR.

In a previous study by Westenberg et al. it was already shown that PWV-assessment from in-plane VE CMR results in a more accurate estimation for global PWV in the complete aorta [21]. Our findings show that also PWV-assessment regionally in the aortic arch as well as locally at the proximal descending aorta correlates better with the gold standard when using in-plane VE CMR instead of through-plane VE CMR. The in-plane PWV-assessment is obtained from a highly dense spatial sampling of the pulse wave along the centerline of the aorta, which contributes significantly to the accuracy.

In this study, the validity of the Bramwell-Hill model was evaluated locally at the proximal descending aorta. This sampling location is technically attractive and pathophysiologically relevant. Motion blurring due to breathing and through-plane motion due to cardiac contraction in the ascending aorta may affect aortic lumen measurements, which are required for distensibility calculations. Respiratory motion will have a similar effect on the lumen assessment both in systole as in diastole. At the location of the descending aorta, the aorta will be subjected to minimal through-plane motion. Furthermore, it is known that the aorta stiffens with age. It was shown that global PWV increases in a nonlinear fashion with age and is most pronounced in the thoracic aorta [28], probably because of cumulative degradation of elastin fibers with age [29] that are most abundant in the thoracic part of the aorta.

To our knowledge, we are the first to test the validity of the Bramwell-Hill model applied at a single location in the aorta. We found a stronger association between PWV, distensibility and pulse pressure for local assessment than for regional assessment. Dogui et al. tested the model on a regional level in the aortic arch and acknowledged the inability to assess local values as a limitation to their study [23]. Nevertheless they found good correlations between regional PWV in the aortic arch and aortic distensibility with correlation still between 0.7 and 0.8. Another discrepancy with the study from Dogui et al. is that in our study the local pulse pressure was assessed invasively and accurately, whereas Dogui et al. only used a global estimation by cuff measurements and carotid tonometry. These practical improvements in our study may possibly explain the higher correlations for the Bramwell-Hill model from our data.

The Bramwell-Hill model can be applied to calculate local PWV-values from distensibility measurements, or vice versa. Theoretically, local pulse pressure can be determined non-invasively from this model, by using local PWV- and aortic luminal distention assessments. Still, in our study a significant underestimation for calculated PWV-values were found when compared to PWV-measurements assessed with invasive pressure. However, this mean underestimation amounted to 0.6 m/s, which may not be clinically relevant since this is in order of 10% of normal values for aortic PWV. Several sources of error might explain this underestimation. First, the CMR and catheterization were not performed on the same day, which is a limitation of our study. The time interval between both examinations might be a possible explanation for the differences, since arterial compliance is subject to physiological day-to-day differences in blood pressure, blood flow and sympathic tone [20]. Also, it is known that beta blocker agents and angiotensin converting enzyme inhibitors may increase aortic compliance and inversely decrease aortic PWV [2,30]. In four patients of the current study population, medication of beta blocking agent was either commenced or increased in dosage between catheterization and CMR. Additionally, in four patients medication with angiotensin converting enzyme inhibitor was initiated or increased. It is unknown what the magnitude of change in aortic PWV will be after changing medication, and what the time interval should be for the medication to have this effect.

Our study has some additional limitations. Automated cuff blood pressure measurements were performed for brachial blood pressure assessment immediately after CMR. The gold standard for measuring brachial blood pressure noninvasively is the mercury manometer. In our study a Dinamap automated blood pressure measurement device was used. It has been described that automated sphygmomanometers are less accurate than mercury manometers. Beaubien et al. showed that only 59% of systolic and 56% of diastolic readings will be within 5 mmHg of the mercury manometer values [31]. Due to practical considerations, the use of an automated sphygmomanometer was required in our study. In order to limit sympathic influences, patients remained in supine position on the CMR-table whilst blood pressure measurements were performed immediately after CMR. Furthermore, this issue is of limited concern since it potentially will introduce a systematic error of equal magnitude on all patients since the same sphygmomanometer was used for all subjects. Furthermore, in the 2006 expert consensus on arterial stiffness it was reported that brachial pulse pressure does not accurately represent aortic pulse pressure [12]. Additionally, blood pressures measurements were obtained invasively during cardiac catheterization, which provide accurate local values of the pulse pressure, necessary for distensibility assessment. Still, the already acknowledged physiological day-to-day differences in blood pressure will restrict the interchangeable use of pressure measurements between examinations. Local invasive pressure should ideally be assessed simultaneously to aortic distention and PWV-assessment. The availability of an interventional CMR environment may facilitate further exploration of the Bramwell-Hill model. Alternatively, this model may also be applied inversely to calculate local pulse pressure from PWV, acquired non-invasively from VE CMR.

Finally, the small sample size is another limitation. This study described a validation of the Bramwell-Hill model for local and regional aortic PWV by VE CMR. No data on the prognostic value of PWV-assessment were presented. Large scale studies, both in patients and healthy volunteers, are needed for further testing the validity of the Bramwell-Hill model and to assess whether local PWV from in-plane VE CMR is superior to regional PWV-assessment for predicting outcome. Additionally, PWV-assessment from in-plane VE CMR has been proven to be accurate and reproducible [21], but image acquisition and analysis still remain time-consuming, which hampers widespread use in clinical practice. Carotid-femoral or sternal-femoral ultrasound data still provide a faster and more cost-effective estimation of PWV.

## Conclusion

CMR with in-plane velocity-encoding is the optimal approach for studying the association between local PWV and aortic distensibility as described by the Bramwell-Hill model. This approach enables local pulse pressure and local distensibility estimation in a non-invasive manner.

## Competing interests

The authors declare that they have no competing interests.

## Authors' contributions

JW is responsible for conception and design of this study, data acquisition, analysis and interpretation of the results and drafting of the manuscript; EvP carried out data analysis, interpretation of the results and drafting of the manuscript; HG carried out data acquisition and revising of the manuscript; PS carried out data acquisition, data analysis, interpretation of the results and revising of the manuscript; WJ carried out data acquisition, interpretation of the results and revising of the manuscript; AdR is responsible for conception and design of this study, interpretation of the results and drafting of the manuscript. All authors read and approved the final manuscript.
